# Prevalence and correlates of *Mycobacterium tuberculosis* infection among healthcare workers at the university hospital in Monterrey, Mexico

**DOI:** 10.1016/j.cegh.2026.102360

**Published:** 2026-04-13

**Authors:** Samvel Gaboyan, Romulo Omar Flores-Perez, Berenice Soto-Moncivais, Brenda Lozano-Rodriguez, Antonino Catanzaro, Marva Seifert, Adrian Rendon

**Affiliations:** a University of California, San Diego, Department of Medicine, Division of Pulmonary, Critical Care, And Sleep Medicine & Physiology, La Jolla, CA, United States; b Universidad Autonoma de Nuevo Leon, Hospital Universitario “Dr. Jose Eleuterio Gonzalez”, Centro de Investigación, Prevención y Tratamiento de Infecciones Respiratorias (CIPTIR), Monterrey, Nuevo Leon, Mexico

**Keywords:** *Mycobacterium tuberculosis* infection, Healthcare workers, TB diagnostics, Occupational health

## Abstract

**Background:**

Healthcare workers (HCWs) are at an increased risk of developing *Mycobacterium tuberculosis* (*Mtb)* infection (TBI) due to occupational exposure to patients with active tuberculosis (TB) disease. The risk is heightened in high TB burden regions. This study aimed to determine the prevalence and conversion rates of TBI among HCWs in a TB treatment hospital in Mexico.

**Methods:**

A prospective cohort study of HCWs (n = 178) was conducted at the public University Hospital of the Universidad Autónoma de Nuevo León (UANL) in Monterrey, Mexico from 2017 to 2019. Eligible HCWs working in the emergency or internal medicine departments were tested at baseline using QuantiFERON^®^-TB Gold Plus (QFT) and Tuberculin Skin Test (TST). Individuals who tested positive were classified as having TBI, while those who tested negative were retested 4–18 months later at a follow-up evaluation.

**Results:**

Prevalence of TBI was 63.5% at baseline as detected by QFT and/or TST. Males, nurses, and administrative staff had increased odds of TBI at baseline. Among the 52 participants who tested negative for TBI at baseline, 16 (30.8%) converted to TBI. Combined baseline and follow-up test results indicated fair agreement (kappa = 0.38) between the QFT and TST.

**Conclusion:**

This study found both high prevalence and conversion of TBI among HCWs at the UANL University Hospital in Monterrey, Mexico and identified associated biological and occupational risk factors. These findings highlight the importance of repeat, short-term testing in regions with high TB prevalence, and underscore the need for improved TBI diagnostics.

## 1. Introduction

Tuberculosis (TB) disease remains a global public health threat. In regions with high prevalence of TB disease healthcare workers (HCWs), individuals who work or train in healthcare settings, face heightened risks of occupational exposure to and infection with *Mycobacterium tuberculosis* (TBI),^1^ which has previously been described as latent TB. The risk of TBI progressing into TB disease is highest during the two years after initial infection,^2,3^ providing a crucial window for case detection and treatment, underscoring the importance of repeat, short-term testing of individuals at highest risk for infection and a better understanding of the factors that contribute to increased risk.

The prevalence of TBI in HCWs varies globally, with studies reporting that in low and middle-income countries, between 33% and 79% of HCWs test positive for TBI.^4^ In Mexico, the prevalence of TB remains notably high among border states, where the incidence rate for TB disease was 44.9 per 100,000 population in 2016, compared to the national rate of 13.8 per 100,000 population.^5,6^ In the border state of Nuevo León, the incidence rate for TB disease was 32 per 100,000 population in 2007–2017, while other studies estimate a prevalence of 28.6% for TB disease in Monterrey, the capital city of Nuevo León, in 2020.^7,8^

The University Hospital of the Universidad Autónoma de Nuevo León (UANL) serves as one of the primary facilities for TB in the region. During 2017 and 2018, 88 and 93 patients were hospitalized for TB at UANL, respectively. In addition, the outpatient TB clinic provided approximately 50 consultations per week to individuals with TB, while the referral laboratory reported over 400 positive TB test results per year, placing HCWs in direct contact with TB patients. Despite the high prevalence of TB disease in Monterrey, estimates of TBI among HCWs in this region are limited to a single study, which reported a prevalence of 20.6% for TBI among medical students only.^9^

Factors including age, sex, body mass index (BMI), and history of vaccination may influence the biological risk of infection, whereas age and job title may shape social and occupational exposure patterns that predispose certain groups to increased risk of infection.^10–15^ Prior studies in Mexico have demonstrated that nurses and older HCWs have increased odds of TBI.^16^ Therefore, we examined these categories of risk to better understand the distinct mechanisms through which infection risk may be mediated in our study population.

Both the QuantiFERON^®^-TB Gold Plus (QFT) and Tuberculin Skin Test (TST) are used to detect TBI. TST has been used traditionally to detect TBI for over a century, however, cross-reactive responses to Bacille Calmette-Guérin (BCG) vaccination have been reported and may lead to false-positive results.^17^ BCG cross-reactivity can be problematic when testing for TB in regions with high rates of BCG vaccination, such as Mexico, where a national vaccination program is in place for BCG. Additionally, TST requires two specifically timed encounters with healthcare personnel to determine a result. QFT only requires a single encounter for testing and results, and does not cross-react with BCG vaccination. However, each test is more expensive than the TST and requires special equipment and personnel, making it a less suitable option for resource-limited regions affected by high burden of TB.^18^ Furthermore, high reversion rates among HCWs following repeat, short-term QFT testing have been reported.^19^ Studies in Nuevo León have identified significant discordance between QFT and TST results,^20^ thus there remains much debate about the appropriate test for repeat, short-term testing.

This observational cohort study aims to identify the prevalence and incidence of TBI among HCWs in Monterrey, Mexico using both QFT and TST; to explore biological and occupational factors associated with baseline TBI and follow-up conversion; and to evaluate the diagnostic compatibility of these tests in this high incidence setting. Results from this study will help inform TB prevention strategies to protect HCWs in high-risk settings.

## 2. Methods

### 2.1. Study population

**Fig. 1.**
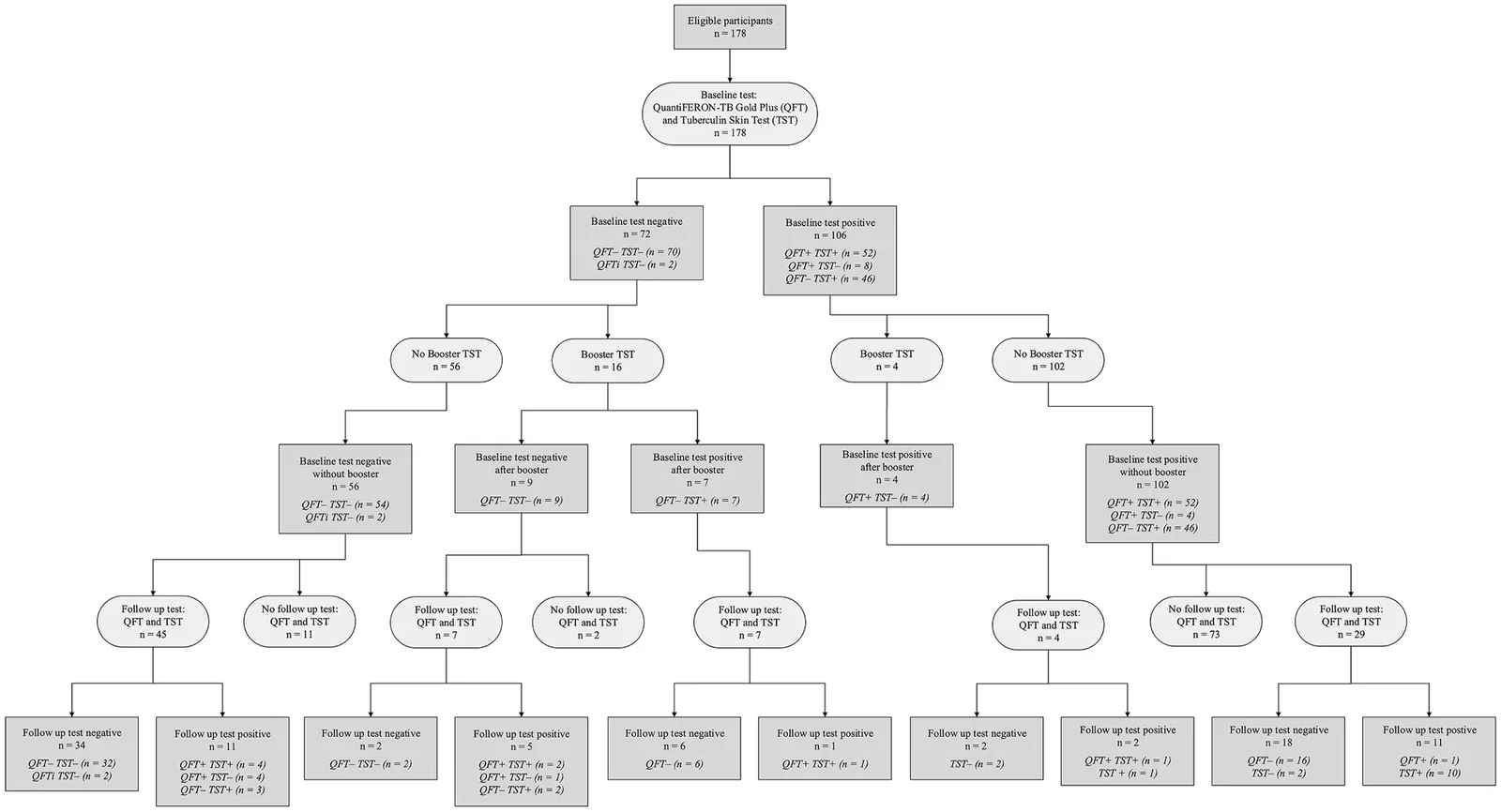
Flowchart of tuberculosis infection (TBI) screening outcomes among healthcare workers at the university hospital in monterrey, Mexico, using QuantiFERON^®^-TB gold plus (QFT) and tuberculin skin test (TST) at baseline and follow-up (n = 178).

This observational cohort study was conducted among employed and student HCWs from the University Hospital of UANL in Monterrey, Mexico. Participants were recruited from March 2017 to May 2019. The prevalence of TBI was assessed using the QuantiFERON^®^-TB Gold Plus (QFT) and Tuberculin Skin Test (TST). Two-step testing was conducted at random among a portion of the participants who tested negative using TST at baseline. Participants with a positive TST booster were considered positive at baseline, whereas those with a negative TST booster were retested using TST at follow-up. Subjects with a negative TST, negative QFT, negative TST and negative or indeterminant QFT at baseline were included in a follow-up evaluation 4–18 months after the baseline testing, during which negative and indeterminant tests were repeated (Fig. 1). Participants who returned a positive TST or QFT result at baseline or during follow-up were referred to the TB clinic for further evaluation. Those who were diagnosed with TBI were provided with a referral to the state TB program for standard of care treatment, 300 mg daily isoniazid for 6 months. This study was approved by the Institutional Review Board of UANL (approval number MI17–00001). Informed consent was obtained from all participants before any procedure was performed.

Our research team recruited HCWs training or working in clinical or administrative roles within the emergency and internal medicine departments at the University Hospital of UANL, and included medical students during clinical training, social service interns, resident physicians, nursing students, employed nurses, and administrative staff. Potential participants were approached directly by study personnel and invited to participate, and if they agreed, were consented and enrolled. Individuals who were <18 years old, had a history of TB disease, had received TBI treatment, or were immunosuppressed (pregnant, malnourished, consumed immunosuppressive drugs, or previously diagnosed with human immunodeficiency virus (HIV) infection or oncologic disease) were excluded. No compensation was provided for participation. The following information was either measured or collected verbally and simultaneously recorded into a written data collection worksheet: sex, age, height, weight, presence of BCG scar, and job title. Body mass index (BMI) was calculated using measured heights and weights and further categorized as underweight if BMI <18.5 kg/ m^2^, normal if BMI <25.0 kg/m^2^ and ≥18.5 kg/m^2^, overweight if BMI <30.0 kg/m^2^ and ≥25.0 kg/m^2^, and obese if BMI ≥30.0 kg/m^2^.

### 2.2. Tuberculin skin test

After demographic information was collected, the Mantoux Tuberculin Skin Test was conducted using the TUBERSOL^®^ (Sanofi Pasteur Limited Toronto, Ontario, Canada) solution. For each subject, 5 TU per 0.1 ml was injected intradermally to the inner surface of the forearm approximately 10 cm below the elbow to create a small bleb. The induration was read by an investigator approximately 72 h after the application. The test was considered positive (+) if the skin induration size was ≥10 mm and negative (−) if it was <10 mm. The 10 mm cut-off for TST positivity reflected Mexico’s national policies, which are aligned with the World Health Organization’s (WHO) criteria for high-risk congregate settings.^21^

### 2.3. QuantiFERON^®^-TB Gold Plus

In addition to the TST test, the QuantiFERON^®^-TB Gold Plus (Qiagen Diagnostics, Hilden, Germany) was used. For each subject, 4 ml of blood sample was drawn via venipuncture of the median cubital vein into four specialized collection tubes (nil, mitogen, TB1, TB2) with 1 ml blood sample in each tube. The tubes were incubated for 16–24 h at 37 °C and centrifuged. Plasma was removed and used to quantify the concentration of released interferon gamma (IFN-γ) via enzyme-linked immunosorbent assay (ELISA). The test was considered positive (+) if IFN-γ concentration of TB1 minus nil or TB2 minus nil≥0.35 IU/ml; negative (−) if IFN-γ concentration of TB1 minus nil or TB2 minus nil<0.35 IU/ml; or indeterminant if nil>8.0 IU/ml or mitogen minus nil<0.5 UI/ml, regardless of TB1 minus nil and TB2 minus nil readings.

### 2.4. Statistical analysis

Descriptive statistics were used to describe characteristics of the participants, and the prevalence and incidence of TBI. Mean and standard deviation (SD) were used to describe continuous variables. Chi-squared and Kruskal-Wallis tests were used to compare proportions and means of categorical and continuous variables, respectively. Logistic regression analysis was used to estimate the odds ratio (OR) and 95% confidence interval (CI) for each variable associated with a positive test at baseline or follow-up. The agreement between paired QFT and TST results was evaluated using Cohen’s Kappa Statistic of interrater agreement, with an almost perfect agreement described by a kappa coefficient value of 0.81–1.00, substantial agreement by 0.61–0.80, moderate agreement by 0.41–0.60, fair agreement by 0.21–0.40, none to slight agreement by 0.01–0.20, and no agreement by ≤ 0.00.^22^ Marginal Homogeneity or difference in classifier performance, was evaluated using the McNamar’s test. A p-value less than 0.05 was considered significant in all tests. All statistical analyses were performed using Stata (version 19.5, StataCorp).

## 3. Results

### 3.1. Characteristics of participants

A total of 178 participants were recruited in this study. Nearly half (46.1%) were medical students in their fourth, fifth, or sixth year. The mean age of the participants was 26 years (SD = 8 years), and 71 (39.9%) were male. At baseline, eight participants (4.5%) were under-weight, and 147 (82.6%) had been vaccinated with BCG according to the national vaccination requirements of Mexico. See Table 1 for the full list of characteristics of all 178 participants.

### 3.2. Prevalence and conversion of TBI

Using a skin induration cut-off size ≥10 mm, 91 participants (51.1%) had a positive TST at baseline prior to booster. Two-step testing was conducted among 20 (11.2%) participants, through which an additional 7 participants had a positive TST booster, yielding a total of 98 participants (55.1%) who had a positive TST at baseline after booster. Using an IFN-γ concentration of TB1 minus nil or TB2 minus nil ≥0.35 IU/ml, 67 (38.1%) patients had a positive QFT at baseline.

Among the 178 participants, 113 were positive for TBI by either or both QFT and TST, with or without booster, yielding a baseline TBI prevalence of 63.5%. Among the 65 participants who tested negative at baseline, 52 (80.0%) were retested at a follow-up evaluation. Among the 52 participants who were retested, 16 were positive by either or both the QFT and TST, yielding a TBI conversion rate of 30.8% over 4–18 months. There were no instances of reversion among the QFT or TST at baseline or follow-up.

Among the 16 participants who converted to TBI, 12 participants were successfully contacted, 11 did not convert to TB disease, and 1 converted to active TB disease. This participant presented with weight loss and diaphoresis, in addition to radiological findings compatible with TB. Following rifampin, isoniazid, pyrazinamide, and ethambutol (RIPE) treatment regimen, the TB symptoms ceased.

### 3.3. Factors associated with baseline infection and follow-up conversion

Among the 34 employed nurses and 13 administrative staff who tested at baseline, 25 (73.5%) and 12 (92.3%) were positive for TBI, respectively. Among the 71 males who tested at baseline, 46 (64.8%) were positive for TBI. The mean age of all the participants who tested positive for TBI at baseline was 27 years (SD = 9 years). After adjusting for age, sex, BMI, BCG vaccination status, and job title, male participants had a 3.0-fold increase(95% CI: 1.3–5.5) in odds of TBI at baseline compared to female participants (see Table 2).

Among the 16 participants who converted to TBI at follow-up, 5 (31.25%) converted to positive on the TST, 5 (31.25%) converted to positive on the QFT, and 6 (37.5%) converted to positive on both tests. The mean age of the 16 participants who converted to TBI at follow-up was 26 years (SD = 5 years), while 12 (75.0%) were female; 6 (37.5%) had normal BMI and 7 (43.8%) were overweight. Among these 16 participants, 15 (93.8%) were vaccinated for BCG, while one had an unknown record of BCG vaccination. Half (50.0%) of the participants who converted to TBI were medical students, and 4 (25.0%) were employed nurses.

After adjusting for age, sex, BMI, and job title, older participants had a 1.4-fold increase in odds of TBI at follow-up (95% CI: 1.0–1.9), while overweight participants had a 7.5-fold increase in odds of TBI at follow-up (95% CI: 1.4–40.6) compared to participants with normal BMI (see Table 3).

### 3.4. Comparison of QFT and TST results

Among the 230 total TST and QFT paired test results, 71 (30.9%) were discordant. The level of agreement between both tests when a QFT cut-off of TB1 minus nil or TB2 minus nil ≥0.35 IU/ml and TST skin induration cut-off of ≥10 mm was fair (kappa = 0.38; 95% CI: 0.25–0.50). Most of the 71 discordant results observed in total were QFT− TST+ (71.8%; 51/71) and thus, were 2.6 times more likely to be QFT− TST + than QFT + TST− (95% CI: 1.5–4.5; see Table 4).

The fair level of agreement between both tests at baseline (kappa = 0.33; 95% CI: 0.19–0.46) was worse than the moderate agreement at follow-up (kappa = 0.49; 95% CI: 0.23–0.76). Most of the 61 discordant results observed at baseline were QFT− TST+ (75.4%; 46/61) and thus, were 3.1 times more likely to be QFT− TST + than QFT + TST− at baseline (95% CI: 1.7–5.9). On the other hand, among the 10 discordant results at follow-up, 5 were QFT− TST+, yielding no significant differences in marginal homogeneity between the two tests at follow-up (see Table 4).

Furthermore, among the TST results, there were no significant associations between positivity and BCG vaccination at both baseline and follow-up (p > 0.05).

## 4. Discussion

### 4.1. Research findings and implications

A baseline TBI prevalence of 63.5% was identified among 178 HCWs at the University Hospital of UANL in Monterrey, Mexico from March 2017 to November 2018. Our findings are comparable to other studies among HCWs rotating and working at hospitals across border states in Mexico, where higher TB rates are reported compared to those in central regions of Mexico.^5,6^ In Chihuahua, 64.5% of physicians, nurses, radiograph and laboratory technicians, administrative staff, and cleaning staff at two general hospitals tested positive in 2001.^16^ In contrast, within the more central regions of Mexico, a lower baseline TBI prevalence of 16.7% was identified among HCWs at a tertiary hospital in Mexico City in 2023.^23^

A follow-up TBI conversion rate of 30.8% was identified among 52 healthcare workers who were retested 4–18 months after a negative baseline result from November 2017 to May 2019. While there are limited estimates on conversion rates of TBI among HCWs in Mexico, our findings are comparable to other studies across Latin America. At a single hospital in Lima, Peru, a region with high TB prevalence, 29.6% of nurses, physicians, administrative staff, and cleaning staff converted to TBI 1 year after negative baseline results.^24^

Our results suggest that male HCWs are 3.0 times more likely to be infected with *Mycobacterium tuberculosis (Mtb)* at baseline compared to female HCWs (95% CI: 1.4–6.4). These results are consistent with an epidemiological investigation conducted at a tertiary hospital in Mexico City, which reported that male HCWs exhibited a two-fold higher likelihood of TBI.^23^ Experimental studies in TB-resistant mice demonstrated that females and castrated males exhibit reduced bacillary burdens and decreased pulmonary inflammation, suggesting that testosterone may be a contributing factor.^10^ In addition, male HCWs have been observed to demonstrate lower compliance with personal protective measures and assigned to tasks associated with greater risk of airborne pathogen exposure relative to their female counterparts.^11^

In addition, we observed 7.5-fold increased odds of TBI conversion among older HCWs (95% CI: 1.0–1.9). Consistent with our findings, older HCWs were reported to be 1.04 times more likely to become infected with *Mtb* at two general hospitals in Chihuahua, Mexico.^16^ Experimental studies in TBI models of mice demonstrated that while younger mice resisted the *Mtb* challenge, older mice developed uncontrolled bacterial growth in lungs due to reduced ability to generate T cells.^12^ Although our study did not capture the duration of employment in healthcare settings, the observed increased odds of conversion to TBI among older HCWs may be attributable to greater cumulative occupational exposure accrued over years of service.

Furthermore, overweight HCWs were identified as being at greater risk for conversion to TBI with a 7.5-fold increase in odds of TBI at follow-up (95% CI: 1.4–40.6) compared to participants with normal BMI. This observation is consistent with findings from a cohort of HCWs in Cali, Colombia, which is a region with high TB prevalence.^25^ Experimental evidence supports this association as well through mice that, when fed with high-fat diets and subsequently infected with *Mtb*, developed increased pulmonary inflammation and bacillary burdens.^15^

A fair agreement (kappa = 0.38) was identified between the QFT and TST among all paired tests in this study, with significant differences in marginal homogeneity and greater odds of testing QFT− TST+. Similarly, one study reported a fair agreement (kappa = 0.39) between QFT and TST among BCG-vaccinated patients in Monterrey, Mexico.^20^ While other studies show that the discordance between these two tests may be driven by TST cross-reactivity with BCG vaccination, our data did not have the power to adequately assess the association between BCG vaccination and TST positivity.^26^ Additionally, discordance between QFT and TST was more pronounced at baseline, which included results from individuals with both recent and historical infection compared to QFT and TST discordance after conversion which included only recently infected individuals. These results suggest that both the QFT and TST may capture true infection; however, the assays may capture biomarkers associated with differing immune responses correlated with infection duration or likelihood of progression to active disease.^27^

### 4.2. Limitations

Our study had some limitations. First, the small number of participants without a BCG scar constrained our ability to assess cross-reactivity between the BCG vaccine and TST. Additionally, evaluating the mechanism of occupational factors on TBI risk was limited due to the lack of data on varying levels of occupational TB exposure and preventive measures practiced by HCWs in our study population. The absence of prospective BMI data posed a limitation as well, since single BMI measurements at baseline may not adequately capture its influence on the risk of conversion. Future directions should include expanding the cohort to capture a larger subset of participants without BCG; implementing an occupational health questionnaire to assess duration and frequency of work history in the healthcare setting, use of protective equipment, and recollections of home-based exposures and encounters with active TB patients; and systematically measuring height and weight at follow-up visits.

### 4.3. Conclusions

In conclusion, using both QFT and TST, this study demonstrated a high baseline prevalence and follow-up conversion to TBI in HCWs in Monterrey, Mexico, and identified biological and occupational risk factors that could better inform targeted preventative measures. These findings suggest that in high TB prevalence regions, repeat, short-term testing is critical for capturing more cases of TBI when the risk for conversion to active TB disease is highest.^2,3^ Furthermore, this study underscored the need for an improved gold standard for the diagnosis of TBI, further justifying the need for additional TB diagnostic development.

## Figures and Tables

**Table 1:** Characteristics of healthcare workers at the university hospital in monterrey, Mexico by baseline QuantiFERON^®^-TB gold plus (QFT) and tuberculin skin test (TST) results for tuberculosis infection (TBI).

Variables	Baseline Tests Results						
	Negative^a^	Positive			Total	p-value	
		Total TBI	QFT + only	TST + only			
N	65 (36.5%)	113 (63.5%)	67 (38.1%)	98 (55.1%)	178		
Age, years^b^	24.3 ± 4.0	27.2 ± 8.8	27.4 ± 9.2	27.8 ± 9.2	26.1 ± 7.5	0.161	
Sex						0.060	
Female	45 (69.2%)	62 (54.9%)	39 (58.2%)	54 (55.1%)	107 (60.1%)		
Male	20 (30.8%)	51 (45.1%)	28 (41.8%)	44 (44.9%)	71 (39.9%)		
BMI						0.121	
Underweight	3 (4.6%)	5 (4.4%)	3 (4.5%)	4 (4.1%)	8 (4.5%)		
Normal	36 (55.4%)	40 (35.4%)	21 (31.3%)	33 (33.7%)	76 (42.7%)		
Overweight	16 (24.6%)	38 (33.6%)	24 (35.8%)	34 (34.7%)	54 (30.3%)		
Obesity	8 (12.3%)	25 (22.1%)	17 (25.4%)	23 (23.5%)	33 (18.5%)		
Missing	2 (3.1%)	5 (4.4%)	2 (3.0%)	4 (4.1%)	7 (3.9%)		
BCG Vaccination						0.609	
Yes	54 (83.1%)	93 (82.3%)	53 (79.1%)	81 (82.7%)	147 (82.6%)		
No	7 (10.8%)	9 (8.0%)	7 (10.4%)	8 (8.2%)	16 (9.0%)		
Missing	4 (6.2%)	11 (9.7%)	7 (10.4%)	9 (9.2%)	15 (8.4%)		
Job Title						0.019	*
Medical Student	40 (61.5%)	42 (37.2%)	25 (37.3%)	30 (30.6%)	82 (46.1%)	0.590	
Fourth Year	6 (15.4%)	5 (12.2%)	4 (16.0%)	2 (6.9%)	11 (13.8%)		
Fifth Year	32 (82.1%)	33 (80.5%)	18 (72.0%)	25 (86.2%)	65 (81.2%)		
Sixth Year	1 (2.6%)	3 (7.3%)	3 (12.0%)	2 (6.9%)	4 (5.0%)		
Social Service	3 (4.6%)	3 (2.7%)	1 (1.5%)	3 (3.1%)	6 (3.4%)		
Resident	8 (12.3%)	23 (20.4%)	13 (19.4%)	20 (20.4%)	31 (17.4%)	0.578	
First Year	6 (100.0%)	17 (73.9%)	11 (84.6%)	14 (70.0%)	23 (79.3%)		
Second Year	0 (0.0%)	3 (13.0%)	1 (7.7%)	3 (15.0%)	3 (10.3%)		
Third Year	0 (0.0%)	1 (4.3%)	1 (7.7%)	1 (5.0%)	1 (3.4%)		
Fourth Year	0 (0.0%)	2 (8.7%)	0 (0.0%)	2 (10.0%)	2 (6.9%)		
Employed Nurse	9 (13.8%)	25 (22.1%)	19 (28.4%)	25 (25.5%)	34 (19.1%)		
Nursing Student	4 (6.2%)	8 (7.1%)	4 (6.0%)	8 (8.2%)	12 (6.7%)		
Administrative Staff	1 (1.5%)	12 (10.6%)	5 (7.5%)	12 (12.2%)	13 (7.3%)		

Note: QFT = QuantiFERON^®^-TB Gold Plus, TST = Tuberculin Skin Test, TBI = Tuberculosis Infection, BCG = Bacillus Calmette-Guérin, BMI = Body Mass Index. Values are shown as frequencies (percent) or means ± standard deviations. Pearson’s chi-squared and Kruskal-Wallis tests were performed to compare variables between participants who tested positive and negative in either test at baseline after booster.

* p < 0.05,

** p < 0.01,

*** p < 0.001.

a Two participants were considered to be negative at baseline because they had negative TST results, but inconclusive QFT results.

b Two participants’ ages were unknown.

**Table 2:** Baseline tuberculosis infection (TBI) by positive QuantiFERON^®^-TB gold plus (QFT) or tuberculin skin test (TST) associated with biological and occupational factors among healthcare workers at the university hospital in monterrey, Mexico (n = 178).

Variables	Bivariate				Multivariate			
	OR	95% CI	p-value		aOR	95% CI	p-value	
Age, years^a^	1.07	1.01 – 1.14	0.020	*	1.03	0.97 – 1.10	0.352	
Sex								
Male	1.85	0.97 – 3.52	0.061		3.01	1.41 – 6.43	0.004	**
Female	ref.	–	–		ref.	–	–	
BMI								
Underweight	1.50	0.33 – 6.73	0.596		1.83	0.36 – 9.32	0.465	
Normal	ref.	–	–		ref.	–	–	
Overweight	2.14	1.02 – 4.47	0.044	*	1.58	0.70 – 3.58	0.275	
Obesity	2.81	1.13 – 7.02	0.027	*	1.85	0.65 – 5.25	0.247	
Missing	2.25	0.41 – 12.32	0.350		2.03	0.33 – 12.39	0.445	
BCG Vaccination								
Yes	1.34	0.47 – 3.80	0.583		1.50	0.40 – 5.64	0.547	
No	ref.	–	–		ref.	–	–	
Missing	2.14	0.47 – 9.70	0.324		2.61	0.44 – 15.59	0.294	
Job Title								
Medical Student	ref.	–	–		ref.	–	–	
Social Service	0.95	0.18 – 5.00	0.954		0.95	0.16 – 5.68	0.957	
Resident	2.74	1.10 – 6.83	0.031	*	1.79	0.64 – 4.99	0.267	
Nurse	2.65	1.10 – 6.35	0.030	*	2.39	0.86 – 6.67	0.096	
Nursing Student	1.90	0.53 – 6.82	0.322		2.14	0.55 – 8.23	0.270	
Administrative Staff	11.43	1.42 – 91.98	0.022	*	9.37	0.89 – 98.46	0.062β	

Note: QFT = QuantiFERON^®^-TB Gold Plus, TST = tuberculin skin test, TBI = tuberculosis infection, BCG = Bacillus Calmette-Guérin, BMI = body mass index, OR = odds ratio, aOR = adjusted odds ratio, CI = confidence interval, ref. = reference group.

* p < 0.05,

** p < 0.01,

*** p < 0.001.

a Two participants were excluded because their ages were unknown.

**Table 3:** Follow-up conversion to tuberculosis infection (TBI) by positive QuantiFERON^®^-TB gold plus (QFT) or tuberculin skin test (TST) associated with biological and occupational factors among healthcare workers at the university hospital in monterrey, Mexico (n = 52).

Variables	Bivariate				Multivariate			
	OR	95% CI	p-value		aOR	95% CI	p-value	
Age, years	1.08	0.93 – 1.24	0.308		1.40	1.03 – 1.90	0.031	*
Sex								
Male	0.47	0.13 – 1.73	0.255		0.45	0.09 – 2.16	0.317	
Female	ref.	–	–		ref.	–	–	
BMI								
Underweight	3.83	0.21 – 70.64	0.366		15.19	0.20 – 1173.26	0.220	
Normal	ref.	–	–		ref.	–	–	
Overweight	4.47	1.09 – 18.37	0.038	*	7.45	1.37 – 40.60	0.020	*
Obesity	0.77	0.07 – 7.86	0.823		0.10	0.00 – 5.29	0.257	
Missing	3.83	0.21 – 70.64	0.366		5.38	0.24 – 121.17	0.290	
Job Title^a^								
Medical Student	ref.	–	–		ref.	–	–	
Social Service	1.50	0.12 – 18.84	0.753		0.46	0.01 – 24.54	0.701	
Resident	1.80	0.35 – 9.28	0.482		1.05	0.11 – 9.68	0.964	
Nurse	3.00	0.61 – 14.86	0.178		0.33	0.03 – 3.96	0.385	

Note: QFT = QuantiFERON^®^-TB Gold Plus, TST = tuberculin skin test, TBI = tuberculosis infection, BCG = Bacillus Calmette-Guérin, BMI = body mass index, OR = odds ratio, aOR = adjusted odds ratio, CI = confidence interval, ref. = reference group. BCG vaccination was not included in this analysis because 7 participants would otherwise be omitted due to perfect prediction of success.

* p < 0.05,

** p < 0.01,

*** p < 0.001.

¶ = One participant was excluded because their age was unknown.

a One participant, an administrative staff member, was omitted because of perfect prediction of failure.

**Table 4:** Marginal homogeneity and interrater agreement between paired QuantiFERON^®^-TB Gold Plus (QFT) and Tuberculin Skin Test (TST) results for tuberculosis infection (TBI) at baseline, follow-up, and total evaluations among healthcare workers at the University Hospital in Monterrey, Mexico.

Evaluations		QFT Test Results			Marginal Homogeneity				Interrater Agreement			
	TST Test Results	Positive	Negative	Total	OR^a^	95% CI^a^	p-value^a^		Coeff.^b^	95% CI^b^	p-value^b^	
Baseline	Positive	52	46	98	3.07	1.68 – 5.91	<0.001		0.33	0.19 – 0.46	<0.001	
	Negative	15	63	78				***				***
	Total	67	109	176								
Follow-up	Positive	8	5	13	1.00	0.23 – 4.35	1.00		0.49	0.23 – 0.76	<0.001	
	Negative	5	36	41								***
	Total	13	41	54								
Total	Positive	60	51	111	2.55	1.49 – 4.52	<0.001		0.38	0.25 – 0.50	<0.001	
	Negative	20	99	119				***				***
	Total	80	150	230								

Note: QFT = QuantiFERON^®^-TB Gold Plus, TST = tuberculin skin test, TBI = tuberculosis infection, OR = odds ratio, CI = confidence interval, Coeff. = kappa coefficient.

* p < 0.05,

** p < 0.01,

*** p < 0.001.

a McNemar’s Test.

b Cohen’s Kappa Statistic.
